# Exploring challenges and coping strategies in providing care for older adults with dementia: a qualitative study conducted in China

**DOI:** 10.3389/fpubh.2025.1459517

**Published:** 2025-02-03

**Authors:** Yanan Cheng, Meijiazi Yang, Sha Ma

**Affiliations:** School of Humanities and Management, Zhejiang Chinese Medical University, Hangzhou, Zhejiang, China

**Keywords:** dementia, older adults, caregivers burden, long-term care insurance, qualitative research

## Abstract

**Background:**

The increasing prevalence of dementias in China has imposed a substantial burden on caregivers, posing a serious threaten to their physical and mental well-being. It is imperative to explore practical measures to alleviate the stress experienced by those caregivers of older adults with dementia.

**Methods:**

A semi-structured questionnaire was adopted for conducting interviews with the participants from December 2022 to January 2023. Subsequently, the interviews were meticulously recorded and transcribed in their entirety, followed by a rigorous analysis of the resulting data and information.

**Results:**

A total of 17 participants who provided care for older adults with dementia were interviewed across various settings including rural areas, communities, hospitals, and nursing homes in Shandong Province. These caregivers encountered challenges such as limited social interaction, significant mental strain, deteriorating physical health, financial insufficiency, and a lack of professional expertise. The current social support systems were deemed insufficient for families caring for older adults with dementia. To alleviate the burden on dementia caregivers, particularly for those with low-income status, the government needs to prioritize enhancing the coverage of the long-term healthcare system and expanding the essential medication list. Given the growing population of older adults in China and the fact that most individuals with dementia are cared for by family members, it is recommended to establish a multifaceted support system involving collaboration among government, social organizations, and volunteers within both urban and rural communities.

**Conclusion:**

The gradual expansion of coverage of long-term care insurance, the proactive inclusion of dementia treatment drugs into the essential medication list, and optimization of a diversified dementia care system ought to be a matter of significant concern.

## 1 Introduction

Due to the rapidly escalating aging population, dementia care has garnered significant attention worldwide. The growing population of older adults with dementia has garnered significant attention in East Asia, particularly in the super-aged nation of Japan. A study predicted that the prevalence of dementia among peopled aged 60 years and older will reach approximately 5.03 million by 2025. In China, which boasts the highest absolute number of older adults globally, providing care for those with dementia presents considerable challenges (1). According to the Seventh National Census of the People’s Republic of China, the population aged 65 and above exceeds 190.64 million, accounting for 13.5% of the total population (2). The aging of the population is a crucial factor contributing to the increase in dementia-related deaths (3). By the year 2050, it is projected that the number of older adults suffering from dementia will reach approximately 27.34 million, accounting for one quarter of the total population of older adults with dementia worldwide (4). The significant increase in the population of older adults with dementia, along with the dramatical changes in the social environment, has implicitly presented greater challenges and heightened standards for caregivers of this vulnerable group. Consequently, public expenditures on long-term care for dementia patients are expected to increase in the future (5).

Older adults with dementia require a greater allocation of healthcare resources, yet the prevailing scarcity of medical facilities and healthcare services in the community has imposed a significant economic burden on caregivers. This burden encompasses not only medical expenses, but also the inability to engage in employment due to enduring both physical and mental health challenges. Consequently, caregivers experience prolonged fatigue and mental stress, which profoundly impacts their personal health and quality of life. “The 14th Five-Year Plan for the Development of the National Aging Cause and the Pension Service System” underscores the importance of strengthening family support policy system for older adults. This study explicitly examines the concept of “respite care” for family caregivers of older adults with dementia. Respite care involves temporarily substituting professional caregivers for family caregivers in caring for older adults with dementia, thereby enabling family caregivers to obtain physical and mental respite.

The caregiving of older adults with dementia poses significant challenges for families in China, a situation that is likely to increasingly affect more households due to the growing population of older adults. While extensive research has been conducted on older adults with dementia, limited attention has been devoted to their caregivers. Therefore, it is imperative to conduct a comprehensive survey addressing the challenges faced by caregivers of older adults with dementia and explore potential solutions. This study aims to (i) delve into the current challenges faced by caregivers of older adults with dementia residing in families, hospitals and pension institutions; (ii) explore potential and feasible countermeasures to alleviate the burden on caregivers of older adults with dementia and enhance their overall quality of life.

## 2 Methods

### 2.1 Participants

Convenient sampling was adopted in this study to select the participants who were caregivers for older adults with dementia residing in homes, hospitals, or nursing facilities within Qingyun County, Dezhou City, Shandong Province. To ensure comprehensive representation, participants were required to meet the following inclusion criteria: (i) having at least 3 months of experience in caring for older adults with dementia; (ii) demonstrating proficiency in effective verbal communication in Mandarin; and (iii) being informed about and voluntarily participating in this study. A total of 17 respondents who provided care for older adults with dementia completed this study, consisting of 8 caregivers residing in home settings (four participants from rural areas and four participants from urban areas), three caregivers from nursing homes, and six caregivers from hospitals.

### 2.2 In-depth interview

A face-to-face in-depth interview lasting approximately 30–60 min was conducted with caregivers of older adults with dementia, residing in homes, hospitals and nursing facilities. To ensure the interviewees can express themselves honestly and without interruption, the locations for interview were meticulously chosen to be quiet, private and welcoming. Prior to each interview session, the researcher introduced herself and provided a comprehensive explanation of the study’s purpose and ethical considerations to the participants. After obtaining consent from the interviewees, formal interviews were conducted by the researcher while recording their responses. A semi-structured interview outline was utilized to collect data on demographic characteristics, caregiving status, challenges encountered during the process of caregiving, and coping strategies employed by caregivers.

### 2.3 Data analysis

After conducting the interview, the researcher accurately transcribed the conversation into Chinese characters according to the original records. Subsequently, the data was meticulously organized and categorized using Excel software, followed by employing the Colizzi analysis method for analyzing the interview data.

## 3 Results

### 3.1 The demographic characteristics of participants

The demographic characteristics of both older adults with dementia and their caregivers are summarized in Table 1. A total of 17 caregivers, aged between 40 and 70 years, who provide care for older adults with dementia (aged between 60 and 89 years) were interviewed. This group comprised 5 males (29.41%) and 12 females (70.59%). Among the older adults with dementia, there were 13 males (76.47%) and 4 females (23.53%). Approximately 88.24% of the older adults with dementia received monthly pensions ranging from 1,000 to 2000 RMB, while about 47.06% of caregivers reported experiencing financial difficulties. Thirteen older adults with dementia resided at home and were cared for by family members, such as spouses and children, whereas four older adults with dementia received care from institutional caregivers.

**Table 1:** The distribution of demographic characteristics among caregivers and older adults with dementia.

Older adults with dementia			Caregivers		
Variables	*n*	%	Variables	*n*	%
Gender			Gender		
Male	13	76.47	Male	5	29.41
Female	4	23.53	Female	12	70.59
Age			Age		
60–70 years	8	47.06	40–50 years	6	35.29
70–80 years	7	41.18	51–60 years	3	17.65
> 80 years	2	11.76	> 60 years	7	41.18
Diagnosed time			Financial situation		
Within 5 years	6	35.29	Poor	8	47.06
5–10 years	6	35.29	General	5	29.41
11–20 years	3	17.65	Good	2	11.76
Over 21 years	2	11.76	Rich	1	5.88
Educational attainment			Relationship with older adults with dementia		
Primary	10	58.82	Spouse	7	41.18
Junior	5	29.41	Child	6	35.29
Senior and above	2	11.76	Others	4	23.53
Pension (RMB)					
1,000–2000	15	88.24			
2001–3,000	1	5.88			
3,001 and above	1	5.88			

### 3.2 The impact of caregiving on the caregiver’s daily life and financial situation

The long-term care for older adults with dementia significantly affects the daily lives of caregivers, particularly regarding social interactions, family finances, and emotional well-being. Approximately 58.8% of caregivers reported that their original social activities have been altered due to their responsibilities toward older adults with dementia. The older adults with dementia required constant attention and companionship, which consumed a significant amount of time and energy from caregivers while isolated caregivers from social interactions. Caregivers always must abruptly end essential social activities out of concern for the safety of the older adults with dementia. The findings of this study revealed that caregivers experienced limitations in their engagement with social activities, resulting in significant changes in both the frequency and breadth of their social interactions.

Furthermore, caregivers of older adults with dementia encounter significant financial challenges. Alarmingly, 11 participants reported that providing care for family members suffering from dementia would plunge the entire household into a severe financial crisis.

*“My husband incurs significant medical expenses annually for hospitalization, injections, and medications. However, the reimbursement process is subject to restrictions based on drug classification and geographical location. Consequently, we are compelled to travel long distances to other regions to purchase medication, but even then, our access is limited. This lack of convenience poses challenges for me as my son resides far away from my domicile and I have restricted availability for medicine procurement. Consequently, I must forego certain drug reimbursement.” (SD-DZ-03, female, home).*

Economic factors played a significant role in the decision-making process for most dementia caregivers. Middle-aged caregivers undoubtedly experience dual pressures as they are required to provide care for both their aged parents suffering from dementia and their own young children simultaneously.

*“Due to my wife’s long-standing dementia condition, she has been on medication for over 20 years, resulting in significant financial burdens. I am unable to engage in employment because I need to provide full-time care for her throughout the day. Our pension is insufficient to cover both medical costs and living expenses, consequently placing a considerable strain on our son who constantly provides us with financial support.” (SD-DZ-06, male, home).*

### 3.3 Caregivers experienced physical and mental health issues

Caregivers of older adults with dementia reported a high susceptibility to physical and mental health deterioration due to long-term, uninterrupted repetitive work and severe emotional exhaustion. A considerable number of caregivers experienced various health issues such as insomnia, lumbar and joint disease, hypertension, diabetes, headaches, anxiety, irritability, depression, powerlessness, and trepidation feelings. Specifically, 12 (70.6%) caregivers experienced dyssomnia, and 9 (52.9%) caregivers reported having at least two health problems. These negative physical health conditions and emotions could be attributed to daily life stressors including financial burdens.

*“I am responsible for providing daytime care duties while also monitoring them at night. However, the situation worsens when some older adults with dementia exhibit nocturnal wandering behavior at nighttime. These abnormal behavioral symptoms caused an excessive noise disturbance, causing significant inconvenience for me. I have experienced countless sleepless nights while providing care for dementia patients, leading to the need for medication to alleviate headaches caused by insomnia. After several years working here, I have developed arthritis and lumbar disease.” (SD-DZ-11, female, nursing facilities).*

*“I have experienced significant stress due to the prolonged caregiving responsibilities for my wife suffering from dementia, which has gravely impacted my physical and mental well-being. I have been diagnosed with hypertension, diabetes, cardiac issues,* var*icose veins, and pain in my arms and legs occasionally rendering me immobile. While family members have helped, they are unable to fully compensate for my role in caring for my wife due to their own work commitments.” (SD-DZ-05, male, home).*

*“I am not good in health due to chronic illnesses such as hypertension and cardiopathy. I consistently experience an irregular heartbeat and elevated blood pressure. Since my father’s diagnosis of dementia, I always carry quick-acting rescue medication for emergency situations. Initially, my older brother and I took turns caring for our father. However, it was inconvenient for him to travel from the city where he works during the period of COVID-19 epidemic. As a result, I dedicate three or four days per week to assist my mother in caring for my father with dementia while balancing work commitments. During periods of busyness, my daughter and son-in-law assume caregiving responsibilities. I will engage a professional nurse if hospitalization is required for serious conditions. Even so, insomnia still occasionally affects me requiring medication.” (SD-DZ-12, female, hospital).*

### 3.4 Limited specialized training and empirical coping strategies

The findings of this study suggested that the primary caregivers for older adults with dementia were predominantly their spouses or children apart from professional workers in nursing facilities. Among the 13 caregivers, only one had received professional training specifically focused on dementia care, while the remaining caregivers possessed no prior experience or formal training in this domain.

*“Neither my children nor I have acquired any specialized knowledge or skills in dementia care. I occasionally scan dementia-related information on platforms such as Douyin or Kuaishou, but I tend to overlook it if it fails to capture my attention. This is primarily because I believed that adequate medication and companionship were sufficient for the care of older adults with dementia. I was unaware of the specific dietary or exercise regimens that could be beneficial for their well-being. My approach has mainly been guided by medical consultations with doctors and my own experiences in daily caregiving.” (SD-DZ-03, female, home).*

*“I have not received any professional training pertaining to dementia caregiving. Instead, I provide care to patients based on the guidance of physicians and the practices demonstrated by nurses.” (SD-DZ-12, male, hospital).*

However, the encouraging aspect is that caregivers have demonstrated a commitment to providing optimal care for older adults with dementia and have implemented various effective strategies to alleviate emotional distress among older adults with dementia, thereby enhancing their quality of life.

*“Despite lacking formal training in dementia care, I have recognized the significance of incorporating physical exercise into my husband’s routine. Considering his personal preference, I bought a pet bird for him to keep at home, enabling him to actively engage with the bird and take it for morning walk. Remarkably, this endeavor consistently yields positive outcomes on his mental state.” (SD-DZ-2, male, home).*

### 3.5 The community dwellers lack adequate social support for dementia care

Participants interviewed in residential and medical settings exhibited lower levels of formal social support, with the majority relying on informal support from personal networks. There was a disparity in dementia care services and information between the government and caregivers of older adults with dementia living in the community. Eleven participants reported being unaware of the caregiving services provided by the government or other social organizations for older adults with dementia. Additionally, many participants mentioned that younger family members were primarily responsible for caring for older adults with dementia. Those experiencing financial difficulties required more support from the government or social organizations, while affluent individuals could afford to employ a caregiver at home.

*“We have not received any specialized dementia care services from the community or social organizations; however, we actively participate in the annual physical examination provided by the Community Health Center. I sincerely hope that financial subsidies for dementia care and assistance in caregiving responsibilities will be available in the future.” (SD-DZ-05, male, home).*

*“Given our family’s financial capability to cover the expenses associated with my father’s dementia. We do not require financial subsidies from the governmental or social organizations. Currently, my mother diligently attends to my father’s daily needs while my brother and I visit frequently. Therefore, we do not immediately require external care support. However, in consideration of long-term care arrangements, I would contemplate hiring a caregiver if my mother’s health deteriorates.” (SD-DZ-15, female, hospital).*

**Figure 1:**
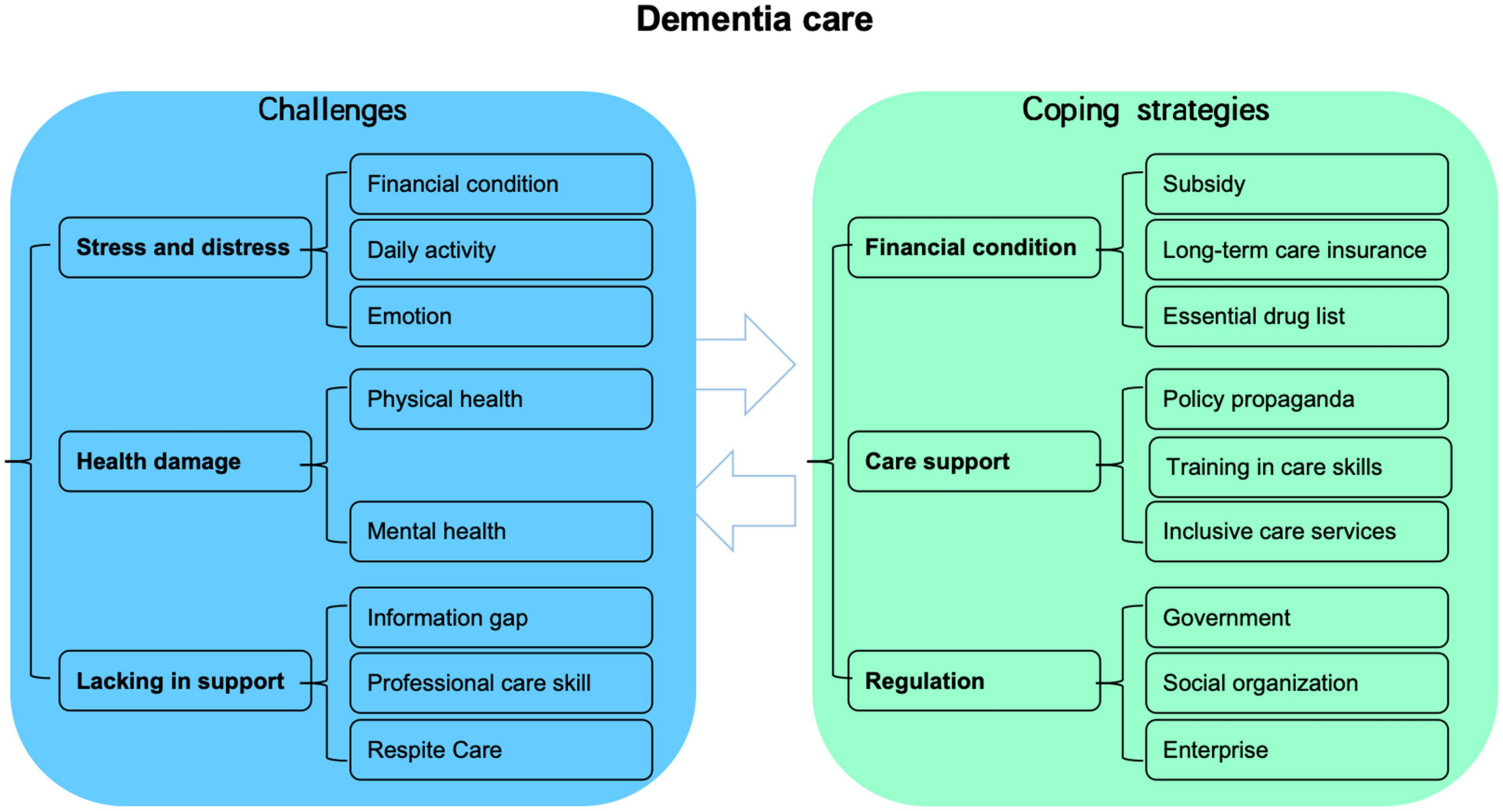
The challenges faced by caregivers of older adults with dementia and the corresponding coping strategies employed to address these difficulties.

Based on the comprehensive data analysis, this study identified significant challenges faced by caregivers of older adults with dementia across various domains including social activities, economics, physical and mental health, as well as social support. To alleviate the burden associated with dementia care, the following remedial measures are recommended for immediate consideration (Figure 1).

## 4 Discussion

### 4.1 Family caregivers of older adults with dementia encounter significant financial challenges

It is estimated that the total annual costs of dementia in China increased from US$0.9 billion in 1990 to US$47.2 billion in 2010, with predictions indicating a surge to US$ 69.0 billion in 2020 and US$ 114.2 billion in 2030 (6). Given the substantial financial implications and the inclination of older adults with dementia. Consequently, the family assumes the primary responsibility for caring for older adults with dementia. It is noteworthy that the majority of care for older adults with dementia is provided by their spouses, children and other family members, aligning with findings from previous research (7).

This study revealed that the primary challenge faced by most families affected by dementia is the considerable financial burden, which encompasses both medical expenses and daily care costs. While health insurance provides partial coverage for the medical expenses, the daily care costs was entirely shouldered by these dementia-affected families themselves. Unless a minority of affluent households are insulated from these financial burdens associated with dementia, the majority experience varying degrees of economic hardship. Essentially, low-income families endure the greatest financial strain, aligning with previous research indicating that such households have limited resources to support dementia care (8).

Undoubtedly, dementia care placed a considerable burden on both families and nations, making the effective integration of resources to achieve mutually beneficial outcomes an essential topic for discussion. For family caregivers, it is particularly urgent to alleviate their financial pressure by improving the dementia-related welfare system, ensuring that individuals with limited resources can afford medical and caregiving expenditure. In addition to pensions and subsidies for the older adults, and it is time to call Chinese government to promptly and comprehensively establish a long-term care insurance (LTCI) system that encompasses provisions for older adults suffering from dementia, while considering the distinct circumstances of various regions. The LTCI was introduced as a pilot program in 2016 across 49 cities in China, showing promising benefits for older adults (9). However, most cases of dementia are currently not covered by the LTCI. Additional research is essential to investigate financing mechanisms and payment standards for LTCI across various regions. Furthermore, it is essential for the government to consider gradually incorporating conventional medications used for dementia to the essential drug list while increasing reimbursement rates, thereby alleviating the financial burdens faced by older adults with dementia and their family caregivers.

### 4.2 Improving dementia care system to alleviate the caregiver’s physical and mental health issues

The findings of this study revealed that many caregivers for older adults with dementia experienced a range of physical or mental health issues, these results were consistent with previous research. A study revealed that 42.8% of primary caregivers for individuals with dementia experienced depression in China (10). Furthermore, the impact of burden on depression among dementia caregivers was intensified by high levels of grief (11). Similarly, this study found that long-term and significant caregiving responsibilities posed a threat to the caregivers’ physical and mental well-being.

Generally, social support plays a crucial role in enhancing the psychosocial well-being of family caregivers, facilitating their caregiving process, and reinforcing their supportive values (12). The health status of caregivers significantly impacts both their own quality of life and that of older adults with dementia. However, we have identified an insufficiency in social support for caregivers of older adults with dementia. While affluent families can afford to employ professional staff in nursing homes or residences, others are left to care for older adults with dementia themselves. This predicament is particularly severe for families with only one child. It is imperative to prioritize and act toward strengthening social support for caregivers of older adults with dementia to alleviate their caregiving burden and ultimately enhance the quality of life for both dementia patients and their caregivers.

Considering the current situation faced by older adults with dementia, it is essential for the government to provide additional support through policy improvements aimed at bolstering dementia caregivers and encouraging interdisciplinary collaboration in the care of older adults with dementia through a variety of innovative approaches. Volunteers can be regarded as invaluable resources in aiding individuals with dementia and their families. Correspondingly, respite care policies should encompass guidelines for eligibility criteria, overall procedures, service standards, and content. In addition to respite care, it is imperative to provide emotional support to dementia caregivers because the prolonged exposure to negative emotions associated with their caregiving responsibilities. Care staff in both urban and rural communities should possess a comprehensive understanding of the specific health information and requirements pertaining to older adults with dementia and their families. Furthermore, nursing facilities and community settings should implement more targeted dementia care services such as psychological assessments for caregivers, psychological counseling, and health education aimed at alleviating emotional stress.

### 4.3 The promotion of dementia education and training for dementia care skills should be enhanced

The present research has identified the emergence of novel challenges. Findings showed that caregiver burden is affected by BPSD (Behavioral and Psychological Symptoms in Dementia), cognition and IADL (Instrumental activities of daily living) (9). Due to a lack of professional technical training, family caregivers often struggle to effectively communicate and interact with older adults suffering from dementia. Consequently, they frequently experience exhaustion from menial tasks and suffer from insomnia, anxiety and other negative emotions. Moreover, many caregivers even develop chronic diseases because of their advanced age and prolonged lack of respite.

Previous study has shown that caregivers lack sufficient knowledge on effectively communication, establishing relationship with, and providing care for individuals living with dementia (13). This study suggests that additional education is required for dementia caregivers to enhance their understanding of dementia care knowledge, caregiving skills, and dementia-related policies beyond the training provided to nursing home staff. Nearly all caregivers reported experiencing exhaustion and emphasized the significance of prioritizing their mental health. Therefore, it is crucial for the government to play a leading role in promoting and implementing policies aimed at supporting family caregivers of individuals with dementia. A specific communication platform consisting of communities, hospitals, and other social organizations should be established to streamline procedures and provide continuous support for these family caregivers. Professional training programs focused on dementia care could be implemented through expert lectures, training courses or peer education opportunities. Additionally, public education campaigns via various media outlets are necessary to reduce social discrimination against those affected by dementia while fostering a more supportive environment within society. Finally, colleges and universities should encourage the development of systematic courses focused specifically on providing relevant professionals with technical expertise in caring for individuals living with dementia.

Considering the diverse needs of dementia care, it is imperative to enhance the integration of a comprehensive social support system encompassing government agencies, family networks, community organizations (rural committee) and social service providers. These entities can provide comprehensive assistance to families affected by dementia, including respite care services, emotional support programs, and auxiliary aid for family caregivers. Access to such services will significantly contribute to the caregivers’ physical and mental well-being.

### 4.4 The establishment of a care system should involve multiple participants

**Figure 2:**
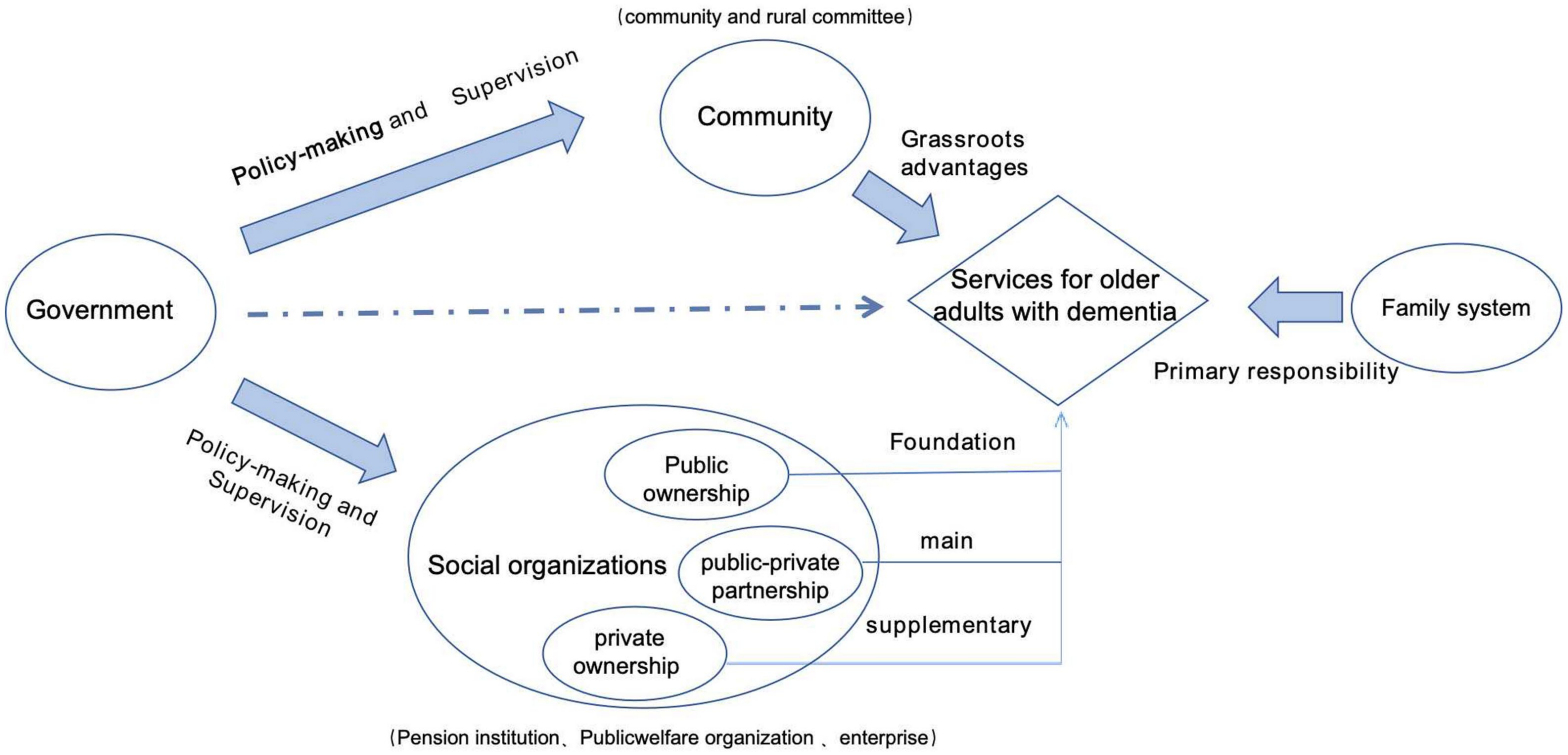
The framework of multiple support systems for dementia care.

The older adults with dementia residing in the community require increased supports due to their low-income condition. The community should be regarded as a platform that offers a wide range of support, integrating multiple stakeholders who can provide diverse services to meet the needs of older adults with dementia and their caregivers (Figure 2).

The government could play a leading and supervisory role in this platform, facilitating the formulation of corresponding legislation and policies, clarifying the roles and responsibilities of all involved stakeholders, ensuring effective operation of dementia care services, as well as regulating and restricting their conduct. It is essential to streamline administration processes, implement decentralization measures, and facilitate collaboration among various care facilities to ensure efficient utilization.

For older adults with dementia living at home, family members assume the primary responsibility for their care. Despite lacking professional training in dementia care, they strive to explore specific and suitable measures for managing the condition. This is because family members, such as spouses or children, possess a unique advantage in providing care for older adults with dementia. In other words, they are familiar with the habits and needs of individuals with dementia and can provide timely feedback that is effective and appropriate, benefiting both their physical and mental health. Family caregivers play a pivotal role in delivering care to older adults with dementia. Given the increasing number of older adults affected by this condition, it is essential to alleviate the challenges faced by dementia caregiver through necessary support services and respite care.

Due to their spatial advantage, grassroots organizations such as community and rural committees played a crucial role as intermediaries between the government and families affected by dementia. Community workers or members of rural committee can effectively address the challenges faced by these families by promoting government policies on dementia care, facilitating access to dementia-related services, and offering respite support for family caregivers.

## 5 Conclusion

Family caregivers of older adults with dementia uncounted numerous challenges related to economic, physical, and mental health issues. Currently, China’s long-term care insurance system has not been fully implemented, and the care for older adults with dementia is not included in the programs of all pilot cities. This situation complicates access to comprehensive support for families caring for those with dementia. Therefore, it is crucial to continuously enhance the long-term care insurance system and gradually improve support services specifically designed for older adults with dementia to alleviate the burden on caregivers of the dementia and enhance their quality of life.

### 5.1 Limitation

The participants were selected using non-probability sampling, which consequently limits the generalizability of the findings to all caregivers of older adults with dementia.

## Data Availability

The raw data supporting the conclusions of this article will be made available by the authors, without undue reservation.
